# PORTEC-4a: international randomized trial of molecular profile-based adjuvant treatment for women with high-intermediate risk endometrial cancer

**DOI:** 10.1136/ijgc-2020-001929

**Published:** 2020-10-12

**Authors:** Anne Sophie V M van den Heerik, Nanda Horeweg, Remi A Nout, Ludy C H W Lutgens, Elzbieta M van der Steen-Banasik, G Henrike Westerveld, Hetty A van den Berg, Annerie Slot, Friederike L A Koppe, Stefan Kommoss, Jan Willem M Mens, Marlies E Nowee, Stefan Bijmolt, David Cibula, Tanja C Stam, Ina M Jurgenliemk-Schulz, An Snyers, Moritz Hamann, Aleida G Zwanenburg, Veronique L M A Coen, Katrien Vandecasteele, Charles Gillham, Cyrus Chargari, Karen W Verhoeven-Adema, Hein Putter, Wilbert B van den Hout, Bastiaan G Wortman, Hans W Nijman, Tjalling Bosse, Carien L Creutzberg

**Affiliations:** 1 Radiation Oncology, Leiden University Medical Center, Leiden, Zuid-Holland, The Netherlands; 2 Radiation Oncology, Erasmus Medical Center - Cancer Institute, Rotterdam, Zuid-Holland, The Netherlands; 3 Radiation Oncology, Maastricht University Medical Centre+, Maastricht, Limburg, The Netherlands; 4 Radation Oncology, Radiotherapy Group, Arnhem, Gelderland, The Netherlands; 5 Radiation Oncology, Amsterdam University Medical Centers, University of Amsterdam, Amsterdam, Noord-Holland, The Netherlands; 6 Radiation Oncology, Catharina Hospital, Eindhoven, Noord-Brabant, The Netherlands; 7 Radiation Oncology, Radiotherapy Institute Friesland, Leeuwarden, Friesland, The Netherlands; 8 Radiation Oncology, Institute Verbeeten, Tilburg, Noord-Brabant, The Netherlands; 9 Women's Health, Universitätsklinikum Tübingen, Tübingen, Baden-Württemberg, Germany; 10 Radiation Oncology, Netherlands Cancer Institute, Amsterdam, Noord-Holland, The Netherlands; 11 Radiation Oncology, University Medical Centre Groningen, University of Groningen, Groningen, Groningen, The Netherlands; 12 Gynecologic Oncology Centre, Department of Obstetrics and Gynaecology, First Faculty of Medicine, Charles University and General University Hospital, Prague, Czech Republic; 13 Radiation Oncology, Haaglanden Medical Center, Den Haag, Zuid-Holland, The Netherlands; 14 Radiation Oncology, University Medical Centre Utrecht, Utrecht, The Netherlands; 15 Radiation Oncology, Radboudumc, Nijmegen, Gelderland, The Netherlands; 16 Women's Health, Rotkreuzklinikum Munchen, Munchen, Bayern, Germany; 17 Radiation Oncology, Isala Klinieken, Zwolle, Overijssel, The Netherlands; 18 Radiation Oncology, Zuidwest Radiotherapeutic Institute, Vlissingen, Zeeland, The Netherlands; 19 Radiation Oncology, University Hospital Ghent, Gent, Oost-Vlaanderen, Belgium; 20 Radiation Oncology, St. Luke's Hospital Dublin, Dublin, Ireland; 21 Radiation Oncology, Institut Gustave-Roussy, Villejuif, Île-de-France, France; 22 Comprehensive Cancer Centre Utrecht, Utrecht, The Netherlands; 23 Medical Statistics, Leiden University Medical Center, Leiden, Zuid-Holland, The Netherlands; 24 Biomedical Data Sciences, Leiden University Medical Center, Leiden, Zuid-Holland, The Netherlands; 25 Obstetrics & Gynecology, University Medical Centre Groningen, University of Groningen, Groningen, Groningen, The Netherlands; 26 Pathology, Leiden University Medical Center, Leiden, Zuid-Holland, The Netherlands

**Keywords:** radiation oncology, endometrium

## Abstract

**Background:**

Vaginal brachytherapy is currently recommended as adjuvant treatment in patients with high-intermediate risk endometrial cancer to maximize local control and has only mild side effects and no or limited impact on quality of life. However, there is still considerable overtreatment and also some undertreatment, which may be reduced by tailoring adjuvant treatment to the patients’ risk of recurrence based on molecular tumor characteristics.

**Primary objectives:**

To compare the rates of vaginal recurrence in women with high-intermediate risk endometrial cancer, treated after surgery with molecular-integrated risk profile-based recommendations for either observation, vaginal brachytherapy or external pelvic beam radiotherapy or with standard adjuvant vaginal brachytherapy

**Study hypothesis:**

Adjuvant treatment based on a molecular-integrated risk profile provides similar local control and recurrence-free survival as current standard adjuvant brachytherapy in patients with high-intermediate risk endometrial cancer, while sparing many patients the morbidity of adjuvant treatment and reducing healthcare costs.

**Trial design:**

A multicenter, international phase III randomized trial (2:1) of molecular-integrated risk profile-based adjuvant treatment (experimental arm) or adjuvant vaginal brachytherapy (standard arm).

**Major inclusion/exclusion criteria:**

Women aged 18 years and over with a histological diagnosis of high-intermediate risk endometrioid endometrial cancer after total abdominal or laparoscopic hysterectomy and bilateral salpingo-oophorectomy. High-intermediate risk factors are defined as: (i) International Federation of Gynecology and Obstetrics stage IA (with invasion) and grade 3; (ii) stage IB grade 1 or 2 with age ≥60 and/or lymph-vascular space invasion; (iii) stage IB, grade 3 without lymph-vascular space invasion; or (iv) stage II (microscopic and grade 1).

**Endpoints:**

The primary endpoint is vaginal recurrence. Secondary endpoints are recurrence-free and overall survival; pelvic and distant recurrence; 5-year vaginal control (including treatment for relapse); adverse events and patient-reported symptoms and quality of life; and endometrial cancer-related healthcare costs.

**Sample size:**

500 eligible and evaluable patients.

**Estimated dates for completing accrual and presenting results:**

Estimated date for completing accrual will be late 2021. Estimated date for presentation of (first) results is expected in 2023.

**Trial registration:**

The trial is registered at clinicaltrials.gov (NCT03469674) and ISRCTN (11659025).

**HIGHLIGHTS**

- PORTEC-4a is the first trial to introduce molecular factors in the adjuvant treatment of endometrial cancer.
- Randomization between standard or individualized treatment based on the molecular risk profile.
- PORTEC-4a will show if omitting treatment in cases of favorable molecular profiles is safe and cost-effective.

## Introduction

The cornerstone of treatment for endometrial cancer is surgery, consisting of a total abdominal or laparoscopic hysterectomy and bilateral salpingo-oophorectomy. Based on clinicopathologic risk factors such as age, stage, histological type, grade, depth of myometrial invasion, and presence of lymph-vascular space invasion, patients are considered to be at low- to low-intermediate risk (approximately 50% of patients), high-intermediate risk (30%–35%), or high risk (15%–20%) for disease recurrence (Table 1).^1^ Adjuvant treatment has increasingly been tailored to these prognostic factors.

**Table 1:** Risk groups of endometrial cancer and current treatment recommendations

Risk group	ESMO-ESGO-ESTRO consensus^1^	Common treatment recommendations
Low risk	Stage I EEC, grade 1–2,<50% myometrial invasion, LVSI negative	No adjuvant treatment
Low-intermediate risk	Stage I EEC, grade 1–2,≥50% myometrial invasion, LVSI negative	Vaginal brachytherapy (consider observation if age <60 years)
High-intermediate risk	Stage I EEC, grade 3, <50% myometrial invasion, any LVSI Stage I EEC, grade 1–2, LVSI unequivocally positive, any myometrial invasion	Vaginal brachytherapy Consider pelvic external beam radiotherapy if LVSI is unequivocally positive, especially if no lymph node dissection or sentinel node has been performed.
High risk	Stage I EEC, grade 3, ≥50% myometrial invasion, any LVSI	External beam radiotherapy Consider vaginal brachytherapy if no LVSI
	Stage II EEC Stage III EEC	Vaginal brachytherapy if grade 1–2 and LVSI negative Pelvic radiotherapy if : Stage II, grade 3 LVSI unequivocally positive Stage III Stage III: combined adjuvant radiotherapy and chemotherapy (PORTEC-3 schedule or sequential)
	NEEC stage I–III (serous, clear cell or undifferentiated cancers; carcinosarcoma)	Vaginal brachytherapy if serous/clear cell, stage IA after full surgical staging, LVSI negative Stage IB–III: combined adjuvant pelvic radiotherapy and chemotherapy

EEC, endometrioid endometrial cancer; ESGO, European Society of Gynecological Oncology; ESMO, European Society for Medical Oncology; ESTRO, European Society; LVSI, lymph-vascular space invasion; NEEC, non-endometrioid endometrial cancer; PORTEC, post operative radiation therapy endometrial cancer.

Multiple randomized trials have established that in stage I endometrial cancer with risk factors, adjuvant external pelvic radiotherapy provides a significant reduction of vaginal and pelvic recurrence. However no overall survival advantage was observed and pelvic radiotherapy is associated with treatment-related toxicities, predominantly gastrointestinal.^2–4^ Since most relapses occurred in the vaginal vault, the PORTEC-2 trial was initiated to evaluate vaginal brachytherapy as compared with pelvic radiotherapy in patients with high-intermediate risk endometrial cancer.^3–5^ The conclusion from the PORTEC-2 trial was that vaginal brachytherapy should be the adjuvant treatment for patients with high-intermediate risk disease because of similar efficacy compared with pelvic radiotherapy, with lower toxicity and better health-related quality of life.^5^ However, adjuvant brachytherapy of all patients with high-intermediate risk features can still be considered overtreatment as there are effective salvage treatments for vaginal relapse in patients who are not previously irradiated.^6^ A long-term analysis of the PORTEC-2 trial showed that a small subgroup of patients with high-risk factors might actually benefit from adjuvant pelvic radiotherapy to maximize pelvic control. These risk factors are substantial lymph-vascular space invasion, L1-Cell Adhesion Molecule (L1-CAM) overexpression, and p53 mutant expression, which are all associated with higher risk of both pelvic and distant relapse.^6–8^

The comprehensive genomic analysis of endometrial cancer by The Cancer Genome Atlas (TCGA) has led to important novel insights in the genomic landscape of endometrial cancer. The TCGA defined four molecular subgroups based on mutational burden and copy number alterations. The ultramutated *POLE*-mutant subgroup is characterized by mutations in the exonuclease domain of *DNA polymerase epsilon* and is associated with an excellent prognosis. The hypermutated subgroup with microsatellite instability (or mismatch-repair deficiency) and the copy-number-low group are both associated with an intermediate (stage-dependent) prognosis. The copy-number-high group, with highly frequent *TP53*-mutations, has an unfavorable prognosis.^9^ Several independent groups have developed and validated surrogate markers for these subgroups that can be assessed on paraffin-embedded tissues. A molecular-integrated risk model was defined by integrating the molecular subgroups with L1-CAM overexpression, substantial lymph-vascular space invasion, and *CTNNB1*-exon 3 mutations.^10–12^ L1-CAM is an independent risk factor of locoregional and distant spread and associated with *TP53*-mutations, high tumor grade, and lymph-vascular space invasion.^12 13^ *CTNNB1*-exon 3 mutations are prognostic for higher risk of recurrence, especially in the copy-number-low group.^11^ By applying this molecular-integrated risk profile to the high-intermediate risk cohorts of the PORTEC-1 and −2 trials, three risk categories (favorable, intermediate, and unfavorable) were defined with a significantly different recurrence-free survival.^11^ It is hypothesized that use of this molecular-integrated risk profile to determine the adjuvant treatment would reduce overtreatment in many patients and reduce undertreatment in a small proportion, with similar vaginal control and recurrence-free survival. The PORTEC-4a trial is the first study worldwide to evaluate the clinical role of molecular risk factors in decision making on adjuvant treatment in patients with endometrial cancer.

## Methods

### Trial design

**Figure 1:**
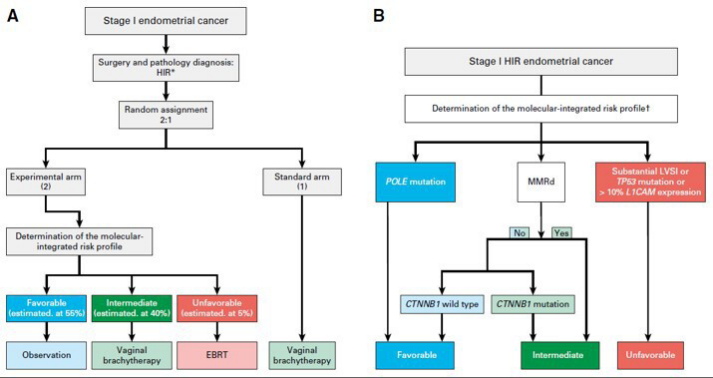
Study design PORTEC-4a trial. Reprinted from 'Molecular-integrated risk profile to determine adjuvant radiotherapy in endometrial cancer: evaluation of the pilot phase of the PORTEC-4a trial' by Wortman et al., 2018, *Gynecologic Oncology* 151; 69–75. A: trial design of the PORTEC-4a trial; B: decision tree for the molecular-integrated profile; *CTNNB1*, β-catenin; EBRT, external beam radiotherapy; LVSI; lymph-vascular space invasion; HIR, high-intermediate risk; L1-CAM, L1-cell adhesion molecule; *POLE*, polymerase-ɛ * stage I (with invasion) disease, grade 3 tumor; stage IB disease, grade 1 or 2 tumor, with either age 60 years or older or substantial LVSI; stage IB disease, grade 3 tumor, without LVSI; or stage II (microscopic) disease, grade 1 tumor.

The PORTEC-4a trial is a prospective, multicenter phase III study, led by the Dutch Gynaecologic Oncology Group. A total of 500 eligible and evaluable women with high-intermediate risk endometrioid endometrial cancer will be randomized (2:1) to the experimental arm with molecular-integrated risk profile-based adjuvant treatment: observation in case of a favorable risk profile, vaginal brachytherapy in case of intermediate risk, and pelvic radiotherapy in case of an unfavorable risk profile; or to the standard arm with adjuvant vaginal brachytherapy. After informed consent and randomization, histopathological slides and a representative tumor tissue block are sent for expert gynecopathology review and determination of the molecular-integrated risk profile. Microscopy is used to confirm histological type, grade and invasion, and, especially, evaluation and semi-quantification of lymph-vascular space invasion. Immunohistochemistry is needed to determine L1-CAM, p53, and mismatch-repair protein expression (MLH1, PMS2, MSH2, and MSH6). Pathogenic *POLE-*mutations and *CTNBB1*-exon 3 status are determined by DNA sequencing. In a small subgroup (3%–6%) of cases the tumor will have more than one classifying feature (combinations of *POLE-*mutations, mismatch-repair deficiency, and p53-abnormal staining).^14^ Risk profiles for these so-called ‘multiple classifiers’ are assigned as follows, based on a previous extensive analysis: favorable in case of a *POLE-*mutation irrespective of other classifiers; intermediate if both mismatch-repair deficiency and p53 abnormal staining, but unfavorable if also substantial lymph-vascular space invasion or L1-CAM overexpression is found. In any other case the least favorable factor will determine the risk profile (see Figure 1).^14^

In each participating country, at least one pathology laboratory has been validated for review and determination of the molecular-integrated risk profile.

Based on previous analysis it is expected that approximately 50%–55% of patients with high-intermediate risk endometrial cancer have a favorable molecular profile.^11^ In the experimental arm this subgroup will receive no adjuvant treatment and will be observed. An estimated 40% of patients have an intermediate profile and will receive vaginal brachytherapy (21 Gy in three fractions of 7 Gy each within an overall time of 2 weeks). About 5%–10% will have an unfavorable profile and receive pelvic radiotherapy (45–48.6 Gy in 1.8–2 Gy fractions) (see Figure 1).^11^ Patients are clinically evaluated during alternating follow-up visits to their gynecologist and radiation oncologist every 3 months for the first 3 years for early detection of local recurrence, and at 6-month intervals until 5 years. For long-term information, vital status and events are also recorded at 7 years. Toxicity is evaluated using Common Terminology Criteria for Adverse Events (CTCAE) version 4.0. and patient-reported health-related quality of life is evaluated using the European Organization for Research and Treatment of Cancer Quality-of-life Questionnaire Core 30 questionnaire (EORTC QlQ-C30)and the endometrial cancer-specific module (EN24). In case of (suspected) recurrence or metastasis, full evaluation is required and treatment with curative intention is initiated if appropriate.

Currently, a total of 22 sites in Belgium, Czech Republic, Germany, France, Ireland, and The Netherlands are open for inclusion and another eight are preparing for participation. The study is supported by the Dutch Cancer Society (UL2011-5336 and UL20).

### Participants

Patients aged 18 years or older with a WHO performance status of 0–2 with a histological diagnosis of endometrioid endometrial cancer with high-intermediate risk features (as listed in Box 1) are eligible after surgery, consisting of a total abdominal or laparoscopic hysterectomy and bilateral salpingo-oophorectomy. Lymphadenectomy, lymph node sampling, or sentinel node procedure are not required but permitted as per center standard protocol.

Box 1 **Inclusion and exclusion criteria of the PORTEC-4a trial**

Inclusion criteria*

- Women aged 18 years and over
- Surgery consisting of a total abdominal or laparoscopic hysterectomy and bilateral salpingo-oophorectomy
- Endometroid endometrial cancer with high-intermediate risk features:
  - Stage IA (with invasion), grade 3 (any age, with or without lymph-vascular space invasion)
  - Stage IB, grade 1 or 2 and age >60 years
  - Stage IB, grade 1 or 2 with documented lymph-vascular space invasion
  - Stage IB, grade 3 without lymph-vascular space invasion
- Stage II (microscopic), grade 1
- Treatment with curative intent

**Exclusion criteria**

- Any diagnosis of malignancy <5 years ago (except non-melanoma skin cancer)
- Previous pelvic radiotherapy
- WHO performance status 3–4
- Expected interval between surgery and start of adjuvant treatment exceeding 8 weeks

*International Federation of Gynecology and Obstetrics stage

Patients diagnosed with malignancy (except for non-melanoma skin cancer) in the past 5 years and/or previous pelvic radiotherapy are excluded. In addition, patients are not eligible if the interval between date of surgery and start of radiotherapy is expected to exceed 8 weeks.

### Primary and secondary endpoints

The primary endpoint is vaginal recurrence. Secondary endpoints are 5-year recurrence-free and overall survival; vaginal control (including treatment for relapse); pelvic and distant recurrence; adverse events; patient-reported symptoms and health-related quality of life; and endometrial cancer-related healthcare costs.

Translational research is an integral part of this molecular-integrated risk profile-based trial. Therefore, a tumor tissue block of all patients is collected and stored in the dedicated tissue bank at Leiden University Medical Center for further analysis.

### Randomization

The study is an open-label phase III trial with (2:1) randomized allocation to either molecular-integrated risk profile-directed adjuvant treatment (experimental arm) or adjuvant vaginal brachytherapy (standard arm). Central randomization is done by a web-based randomization application with stratification for participating center, tumor grade (1 vs 2 vs 3), and type of surgery (lymphadenectomy yes or no) using a biased coin minimization procedure. The trial number and result of randomization are immediately obtained via the randomization system and confirmed by email.

### Sample size

It is hypothesized that the experimental treatment strategy leads to less overtreatment, which would yield less morbidity and a better quality of life. As treatment will be deescalated in a substantial part of the patients in the experimental arm, a non-inferiority design was chosen to estimate vaginal recurrence in both groups with sufficient precision and to exclude a clinically relevant difference with an equivalence margin of 7%. Other first recurrences (pelvic and distant) and death are considered competing risks. Based on data from the PORTEC-2 trial and the molecular analysis from the pooled PORTEC-1 and 2 trials, the expected 5-year rates of vaginal recurrence are 2.0% in the vaginal brachytherapy arm and 4.625% in the molecular profile arm. The null hypothesis states that the 5-year cumulative incidence of vaginal recurrence in the molecular profile-directed treatment arm exceeds that of the standard vaginal brachytherapy arm by more than an equivalence margin of 7%: the null hypothesis will be rejected if the upper bound of the one-sided 95% Wald confidence interval of the difference (molecular arm minus standard arm) does not exceed the equivalence margin. The inclusion period was estimated to be 4 years, with a follow-up period of 3 years after inclusion of the last patient. For a total of 500 evaluable patients, with 167 patients in the standard arm and 334 in the molecular arm, a power of 84.4% is achieved. An additional power calculation was performed for a subgroup analysis among all patients with a favorable risk profile to determine the difference in vaginal recurrence between the experimental group (no adjuvant treatment) and the standard treatment group (brachytherapy). These groups will be compared using a non-inferiority design with an equivalence margin of 8.5%. The power will depend on the actual difference between the arms. For example, if the 5-year cumulative incidence of vaginal recurrence would be 4% in the experimental arm and 1.5% in the standard arm, the power will be 89.7%: for 5% vs 1.5% this would be 75%. The main aim of this analysis is to estimate the difference in vaginal recurrence in the favorable subgroups with sufficient precision (SE of the difference below 2.4%).

The pilot phase (first 50 patients) of the PORTEC-4a trial has been completed and showed that the trial design is acceptable for patients and feasible in terms of the logistics for determining the molecular profile within 2 weeks.^13^ Currently, 366 patients have been included. Monitoring of safety is done by the Data and Safety Monitoring Board. Annual safety reviews of serious adverse events, deaths, and recurrences are presented confidentially to the Data and Safety Monitoring Board, which is free in its recommendations to the study coordinators and confidential recommendations to the study statistician.

### Statistical methods

All analyses concerning treatment effects will be performed according to the intention-to-treat principle. Time-to-event analyzes will be performed with date of randomization as a starting point. Formal tests for the differences in relapse and survival rates between the two arms will be done with the Kaplan–Meier method, the log-rank test, and Cox regression analysis. The competing risk method will be used, with competing risks of death, pelvic recurrence, and distant recurrence for analysis of vaginal recurrence, and competing risk of death for analyses of pelvic and distant recurrence. Analysis of toxicity will be based on treatment received. P-values<0.05 will be considered statistically significant.

For the cost-utility analysis, endometrial cancer-related healthcare costs will be considered. This includes in both arms of the trial the costs of: primary treatment, care associated with adverse events, and first treatment for relapse and/or metastasis. Healthcare use across the follow-up period will be converted to costs using standard prices, discounted over time. The difference in quality-adjusted life years and costs between the two arms will be compared according to the intention-to-treat principle, using *t*-tests with, if appropriate, multiple imputation of missing data.

Health-related quality of life analysis will be performed for patients with a valid baseline measurement and at least one follow-up measurement. To evaluate the differences between the treatment groups with respect to the effect of treatment burden on life quality during and up to 5 years after treatment, the repeated measures of the scales of the quality of life questionnaires will be analyzed using mixed ANOVA models. Single items will be analyzed using logistic regression at different timepoints.

## Discussion

Several randomized trials, such as the GOG-99, ASTEC, and PORTEC-1 and −2 trials, have established recommendations for the adjuvant treatment of endometrial cancer based on clinicopathological risk factors.^2–5^ Current international guidelines recommend adjuvant vaginal brachytherapy for women with early stage high-intermediate risk endometrial cancer, while those with low- and low-intermediate risk disease are recommended to be observed after surgery.^1–5^ The molecular classification of endometrial cancer, as first defined by TCGA and subsequently validated by the use of surrogate markers which makes the molecular tests available in daily clinical practice, has profoundly altered the risk classification. The PORTEC-4a trial is the first randomized trial to implement the use of molecular factors to determine adjuvant treatment. The molecular-integrated risk profile includes both the molecular groups and other essential risk factors such as substantial lymph-vascular space invasion and L1-CAM overexpression.^7 12^ The PORTEC-4a trial will provide essential information on whether vaginal brachytherapy can be safely omitted in patients with a favorable molecular-integrated risk profile, and whether patients with an unfavorable risk profile would benefit from more intensive adjuvant treatment. If local control rates in the molecular-integrated risk profile-directed adjuvant treatment arm are not significantly worse than in the standard arm, then patients will have the benefit of less overtreatment, with an expected substantial reduction in morbidity and a better quality of life.

The acceptance and implementation of the molecular-integrated risk profile in standard care will be facilitated if the cost-utility analysis shows that molecular-integrated risk profile-tailored adjuvant treatment leads to similar or even reduced healthcare costs. Even though the costs in the experimental group will be higher due to determination of the molecular-integrated risk profile by next-generation sequencing, about 50%–55% of the patients with a favorable profile will receive no adjuvant treatment which will substantially reduce costs. However, the costs of any additional recurrence in the experimental arm may partially counterbalance this effect. In addition, it is expected that a substantial reduction in the cost of the molecular profile will be realized as cheaper targeted *POLE* sequencing methods are being developed.

During the trial both national and international pathology laboratories are being validated for determination of the molecular-integrated risk profile, leading to early and rapid dissemination and optimization of the molecular techniques and logistical processes. If cost-utility is confirmed, it is expected that molecular-integrated risk profile-directed adjuvant treatment will rapidly be accepted as the standard approach in clinical practice and used in future studies.
